# Motoneuronal Spinal Circuits in Degenerative Motoneuron Disease

**DOI:** 10.3389/fnmol.2020.00074

**Published:** 2020-05-25

**Authors:** Mélanie Falgairolle, Michael J. O’Donovan

**Affiliations:** Section on Developmental Neurobiology, National Institute of Neurological Disorders and Stroke, National Institutes of Health, Bethesda, MD, United States

**Keywords:** locomotion, spinal muscular atrophy, amyotrophic lateral sclerosis, central pattern generator, recurrent collaterals

## Abstract

The most evident phenotype of degenerative motoneuron disease is the loss of motor function which accompanies motoneuron death. In both amyotrophic lateral sclerosis (ALS) and spinal muscular atrophy (SMA), it is now clear that dysfunction is not restricted to motoneurons but is manifest in the spinal circuits in which motoneurons are embedded. As mounting evidence shows that motoneurons possess more elaborate and extensive connections within the spinal cord than previously realized, it is necessary to consider the role of this circuitry and its dysfunction in the disease process. In this review article, we ask if the selective vulnerability of the different motoneuron types and the relative disease resistance of distinct motoneuron groups can be understood in terms of their intraspinal connections.

## Introduction

Degenerative motoneuron diseases are devastating conditions whose underlying causes are poorly understood. Two of these diseases, amyotrophic lateral sclerosis (ALS) and spinal muscular atrophy (SMA), result in loss of motoneurons, leading to reduced motor function and ultimately death (Cleveland and Rothstein, 2001; Wee et al., 2010). Although both disorders have been considered autonomous motoneuron diseases, it is now clear that their pathology is not restricted to motoneurons and that dysfunction is more widespread, particularly within the brainstem and spinal circuits in which the motoneurons are embedded (Schütz, 2005; Ling et al., 2010; Mentis et al., 2011). Because of this, the primary, cell-autonomous pathologies caused by the conditions are compounded by secondary effects that result from disruptions in the spinal circuitry. The resulting motor deficits are, therefore, due to the interactions between the primary and secondary processes.

In this review article, we will first discuss the spinal circuit abnormalities in both SMA and ALS. We will then describe the sensitivity of the different motoneuron types to the diseases, and finally, we will consider these differences in susceptibility in light of recent discoveries showing that the intraspinal connections of motoneurons are more extensive than previously appreciated (Bhumbra and Beato, 2018; Chopek et al., 2018). These new connections create the likelihood of additional secondary effects that will further complicate interpretation of the disease process. We will conclude the review by considering these novel findings in relation to the motor dysfunction, and we will ask if the known susceptibility of different motoneuron types and different motoneuron pools to disease can be understood by considering differences in their intraspinal connectivity.

## Motoneuronal Circuits in Degenerative Motoneuron Disease

The SMNΔ7 mouse model of SMA lacks the SMN gene but expresses two copies of the human SMN2 gene (Le et al., 2005). These mice exhibit several motor defects, including weakness and an inability to right themselves, and they eventually die at 2 weeks of age. The proximal muscles are more affected than the distal muscles, with the epaxial and hypaxial muscles being the most severely weakened (Montes et al., 2009; Mentis et al., 2011). One of the first pathological changes in the disease is a decline in the strength of muscle spindle afferent synaptic input to motoneurons (Ling et al., 2010; Mentis et al., 2011; Fletcher and Mentis, 2017; Fletcher et al., 2017). This loss of muscle afferent input to motoneurons is due to a decrease in the amount of glutamate released from the afferents onto motoneurons (Fletcher et al., 2017). In addition to a loss of proprioceptive input to motoneurons, there is a reduction in the number of vesicular glutamate transporter (VGLUT)2^+^ terminals on motoneurons in SMNΔ7 mice (Ling et al., 2010) that can be derived from local (Ling et al., 2010) or descending (Du Beau et al., 2012) glutamatergic interneurons. Loss of somatic vesicular gamma aminobutyric acid (GABA) transporter (VGAT) terminals was not observed, suggesting that the inhibitory inputs to motoneurons are less affected in the disease than excitatory inputs (Ling et al., 2010).

The decreased glutamate output from primary muscle spindle afferents triggers several changes in the properties of motoneurons, including an increase in input impedance and a downregulation of the Kv2.1 potassium channel (Fletcher et al., 2017). These responses are probably compensatory because they also occur in wild-type mice in which transmitter release from muscle afferents is abrogated by tetanus toxin (Fletcher et al., 2017). Proof of the secondary nature of the altered motoneuron electrical properties in SMA comes from experiments in which the SMN protein was selectively restored in afferents or motoneurons (Fletcher et al., 2017). Restoration of the protein in afferents, but not in motoneurons, normalized Kv2.1 expression and partially restored the firing of motoneurons to current injection (Fletcher et al., 2017). These findings illustrate that the pathology exhibited in SMA is a combination of cell-autonomous abnormalities, secondary changes due to the interaction of motoneurons with abnormally functioning afferents, and the compensatory responses of both motoneurons and afferents to their primary and secondary defects (Brownstone and Lancelin, 2018). Although secondary, the motoneuronal changes contribute significantly to the motor deficits in SMA.

If the reduced synaptic input from primary afferents to motoneurons reflected generalized afferent dysfunction and was independent of motoneuron pathology, then we would predict that afferent loss should also be observed on other intraspinal targets of primary afferents. This idea was tested by examining the number of VGLUT1 primary afferent terminals on Renshaw cells (RCs) in SMNΔ7 neonatal mice (Thirumalai et al., 2013). However, in contrast to the findings in motoneurons, the number of VGLUT1 terminals on RCs was increased rather than decreased. While this suggests that not all branches of proprioceptive afferents exhibit the same fate, it is not known if the proprioceptive synapses on RCs are functional. The cause of this increased afferent innervation is unknown, but one possibility is that primary afferents sprout in response to the loss of inputs to motoneurons. Interestingly, the number of cholinergic vesicular acetylcholine transporter (VAChT)^+^ terminals was also increased onto RCs in the rostral lumbar segments at P13 even though there was a substantial loss of motoneurons in these segments. Again, the mechanisms responsible for this are unclear, but it may also represent sprouting because the remaining motoneurons will have lost a significant portion of their motoneuronal targets (Nishimaru et al., 2005; Bhumbra and Beato, 2018). The consequences of these changes in connectivity within motor circuits are not known. An increased innervation of RCs, if functional, could serve to inhibit motoneuron firing, thereby exacerbating the weakness exhibited by these animals.

There are several mouse models of ALS, but here we will concentrate on the superoxide dismutase (SOD)1 G93A model because most work has been done using this line (Rosen et al., 1993). Unlike the SMNΔ7 model of SMA, the natural history of the disease in the SOD1 G93A mouse is much more prolonged with animals living to 150 days. Furthermore, in contrast to the findings in SMA, inhibitory spinal circuits exhibit abnormalities early in the disease. Some of these changes can be detected even before birth. For example, in SOD-93 mice, the GABA equilibrium potential recorded in motoneurons is more depolarized than in wild-type animals, indicating an alteration in chloride homeostasis at E17.5 (Branchereau et al., 2019). At this early stage, there is also a deficiency of inhibitory synaptic terminals on motoneurons which persists into postnatal life (Martin and Chang, 2012; Branchereau et al., 2019). Studies of cultured motoneurons and interneurons showed that glycine currents are smaller in motoneurons from the mutant mice compared to their wild-type counterparts. The loss of glycinergic function appears to be specific for large motoneurons because it is not observed in presumed gamma and small, fatigue-resistant (S-type) motoneurons that innervate type I muscle fibers (Chang and Martin, 2011). The reduced inhibitory input could be due to loss of inhibitory interneurons or to weaker inputs from inhibitory neurons (Chang and Martin, 2009; Wootz et al., 2013). Consistent with the latter idea, Wootz et al. (2013) showed that the innervation of RCs by motoneurons was lost at early stages of the disease and was associated with a downregulation of VAChT in motoneurons. Eventually, a majority of the motoneuronal synapses on RCs are lost. It seems likely that the synaptic projections of motoneurons to other motoneurons and to V3 interneurons will also be lost at some stage in the disease.

Muscle spindle afferent inputs to motoneurons are also affected in the SOD1 mouse model of ALS. VGLUT1 immunoreactivity, presumed to originate from proprioceptive afferents, is reduced in the motor nucleus at day 110 and is almost absent at day 130, indicating loss of muscle spindle afferent input to motoneurons (Schütz, 2005). This was confirmed by Vaughan et al. (2015) who showed that proprioceptive nerve endings initially degenerate in the periphery, and this is followed by loss of their central projections onto motoneurons. Electrophysiological studies of monosynaptic afferent connections in sacral motoneurons have shown that the evoked response recorded from the ventral roots declines with age, and although this was attributed to a loss of motoneurons (Jiang et al., 2009), it seems likely that it also reflects loss of proprioceptive input to motoneurons. Proprioceptive afferents in the mesencephalic nucleus of the SOD1 mouse exhibit reduced excitability at P11 due to reduced expression of Nav1.6-type Na^+^ currents, which could lead to compensatory increases in the excitability of their target motoneurons (Seki et al., 2019).

## Motoneuron Classes and Their Susceptibility to the Disease Process

Degenerative diseases do not affect all motoneuron classes uniformly. For instance, in both SMA and ALS, the motoneurons innervating the extraocular muscles and the anal and bladder sphincters are spared (Comley et al., 2016; Nijssen et al., 2017). In this section, we consider the different motoneuron types and ask if their susceptibility to the disease differs and whether this is correlated with any features of their intraspinal circuitry.

In mammals, motoneurons innervating skeletal muscles comprise three classes (for a review, see Manuel and Zytnicki, 2011): α-motoneurons that innervate the extrafusal fibers, γ-motoneurons that innervate intrafusal muscle fibers (Kuffler et al., 1951), and β-motoneurons that innervate both (Bessou et al., 1963). α-Motoneurons can be further subdivided into fast-twitch fatigable (FF) motoneurons that control type IIb muscle fibers, fast-twitch fatigue-resistant (FR) motoneurons that control type IIa muscle fibers, and slow (S) motoneurons that control type I muscle fibers (Burke et al., 1971). There are two types of γ-motoneurons: static type that innervates the bag2 and chain fibers of the spindle, and dynamic type that innervates the bag1 fiber of the spindle (Matthews, 1963; Brown et al., 1965). Static β-motoneurons innervate type II extrafusal muscle fibers and the bag 2 and the chain fibers of the muscle spindle, whereas the dynamic β-motoneurons preferentially innervate type I skeletal muscle fibers and the muscle spindle bag1 fiber. β-Motoneurons innervate from 30% to 70% of the muscle spindles (McWilliam, 1975) and constitute from 11% to 30% of the axons supplying the extrafusal muscle fibers (Emonet-Dénand and Laporte, 1975; McWilliam, 1975). In both ALS and SMA, the largest motoneurons (FF) are the most vulnerable, followed by the FR, with the S motoneurons being the last to degenerate (for a review, see Kanning et al., 2010). While γ-motoneurons are resistant to ALS and SMA, β-motoneurons appear to be as vulnerable to the disease as α-motoneurons (Lalancette-Hebert et al., 2016; Powis and Gillingwater, 2016).

In addition, the external anal sphincter of the cat (innervated by motoneurons in Onuf’s nucleus) is a slow twitch muscle (Bowen and Bradley, 1973) that is presumably innervated by type S motoneurons. This might explain some of the resistance of these motoneurons to disease. In contrast, the extraocular muscles comprise six different types of muscle fiber including slow- and fast-twitch fibers and multiply-innervated non-twitch fibers (Evinger et al., 1979; Yu et al., 2005; Nijssen et al., 2017), suggesting that the resistance of the motoneurons to disease is not explained by the types of muscle fiber they innervate, consistent with transplant studies between SOD1-G93A and wild-type mice (Carrasco et al., 2010).

Many explanations have been proposed to account for the differences in the susceptibility of motoneurons to disease pathology. Here, we focus on the synaptic inputs and outputs of motoneurons innervating the hind limb and ask if any aspect of this connectivity is correlated with the susceptibility of the different motoneuron types to disease.

## Synaptic Inputs to Motoneurons

One difficulty in drawing general conclusions is that the work on motoneuron connectivity has been done in different species at different ages and on a relatively limited set of motoneuron pools. Electron microscopy studies in cat have shown that α-motoneurons have four main types of boutons (McLaughlin, 1972b; Conradi et al., 1979a; Brännström, 1993): S-type boutons (small diameter with spherical synaptic vesicles), F-type boutons (small diameter with flattened synaptic vesicles), C-type boutons (large diameter with subsynaptic cisterns), and M-type boutons (large diameter that disappear after dorsal root section). The S-type boutons have been associated with excitatory synapses, the F-type with inhibitory synapses (Uchizono, 1965; Brännström, 1993), M-type with afferent inputs (McLaughlin, 1972a), and C-type with cholinergic inputs arising from V0c neurons expressing the pituitary homeobox (PITX)-2 transcription factor (Hellström et al., 2003; Zagoraiou et al., 2009). Ia afferent synapses are either S-type (Fyffe and Light, 1984) or the larger M-type that are apposed to P-type presynaptic boutons (Ornung et al., 1995).

The different motoneuron classes do not receive the same number or type of inputs. In particular, C-boutons are much more frequent on F-type motoneurons than on S-type motoneurons (Conradi et al., 1979b; Kellerth et al., 1979; Brännström, 1993; Hellström et al., 2003), and the number of Ia synaptic contacts is higher on FF motoneurons than on the S-type (Burke and Glenn, 1996). γ-Motoneurons appear to lack C-boutons and Ia contacts in cats and rodents and have less diversity in their synaptic inputs than α-motoneurons with only S- and F-type boutons on their proximal dendrites and their cell bodies lacking the M-, a C-type synapse found on α-motoneurons (Arvidsson et al., 1987; Simon et al., 1996; Ichiyama et al., 2006).

**Figure 1:**
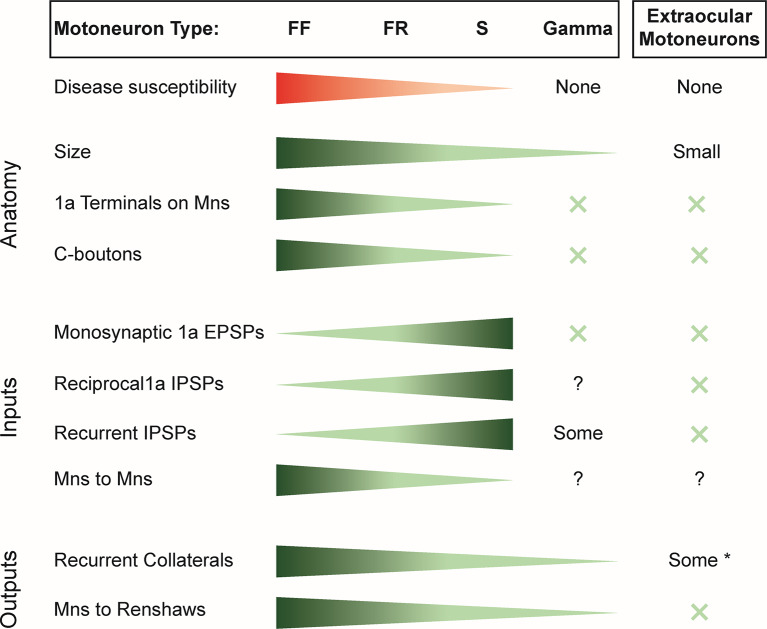
Summary of the susceptibility of motor unit types to spinal muscle atrophy (SMA) and amyotrophic lateral sclerosis (ALS) and the distribution of inputs and outputs of the different motoneuron classes. X indicates the absence of the variable considered. *Note that only some extraocular motoneurons have recurrent collaterals; others have none.EPSP, excitatory postsynaptic potential; IPSP, inhibitory postsynaptic potential; FF, fast-twitch fatigable motoneuron; FR, fast-twitch fatigue-resistant motoneuron; S, slow motoneuron.

A review of literature reveals that the synaptic efficacy of the inputs to motoneurons, measured as the size of the excitatory postsynaptic potential (EPSP) or inhibitory postsynaptic potential (IPSP), is generally highest in the type S motoneurons followed by the type FR and then the type FF (Figure 1). This is true for the monosynaptic inputs from muscle spindle afferents (Burke and Rymer, 1976) even though the number of afferent synaptic contacts on motoneurons exhibits the reverse distribution (Burke and Glenn, 1996). The discrepancy between the distribution of synaptic efficacy and the number of afferent terminals reflects differences in the input impedance of the different motoneurons, with the highest in type S and the lowest in type F. The synaptic efficacy of inhibitory inputs, including disynaptic 1a inhibition (Burke and Rymer, 1976) and recurrent inhibition (Hultborn et al., 1988), is also highest in type S motoneurons and weakest in type FF motoneurons. In contrast, γ-motoneurons lack monosynaptic primary afferent inputs but do receive polysynaptic excitatory and inhibitory inputs from other afferents (Eccles et al., 1960; Appelberg et al., 1983a,b,c) as well as inhibition from RCs (Ellaway and Murphy, 1981; Appelberg et al., 1983d).

Thus, within the α-motoneuron population, there is an inverse relation between the strength of synaptic inputs and the susceptibility of the different motoneuron types to disease. Accordingly α-motoneurons have the largest inputs from muscle spindle afferents, 1a inhibitory interneurons, and recurrent inhibition. However, this correlation fails when the disease-resistant, γ-motoneurons and extraocular motoneurons are considered because they receive only weak or no input from these synaptic sources (Figure 1).

## Synaptic Outputs of Motoneurons and Their Effects on Spinal Circuits

The best described output connection of motoneurons is to the inhibitory Renshaw cell population (Renshaw, 1946; Eccles et al., 1954, 1961). In the adult cat, it has been estimated that the largest inputs to RCs are from FF motoneurons with progressively fewer from FR and type S motoneurons (Hultborn et al., 1988). The distribution of inputs from motoneurons to RCs appears to reflect the number of collateral swellings, presumed to be presynaptic terminals, which is greatest on the type FF motoneurons followed by FR and S (Cullheim and Kellerth, 1978). In addition, γ-motoneurons have very few recurrent collaterals (Cullheim and Ulfhake, 1979; Westbury, 1982). It was also known from work in adult cats that α-motoneuron recurrent collaterals project to other α-motoneurons irrespective of their motor unit type (Cullheim et al., 1977, 1984). However, at least in the adult cat, it is not clear that these are functional because no reports have described excitatory synaptic connections between feline motoneurons.

**Figure 2:**
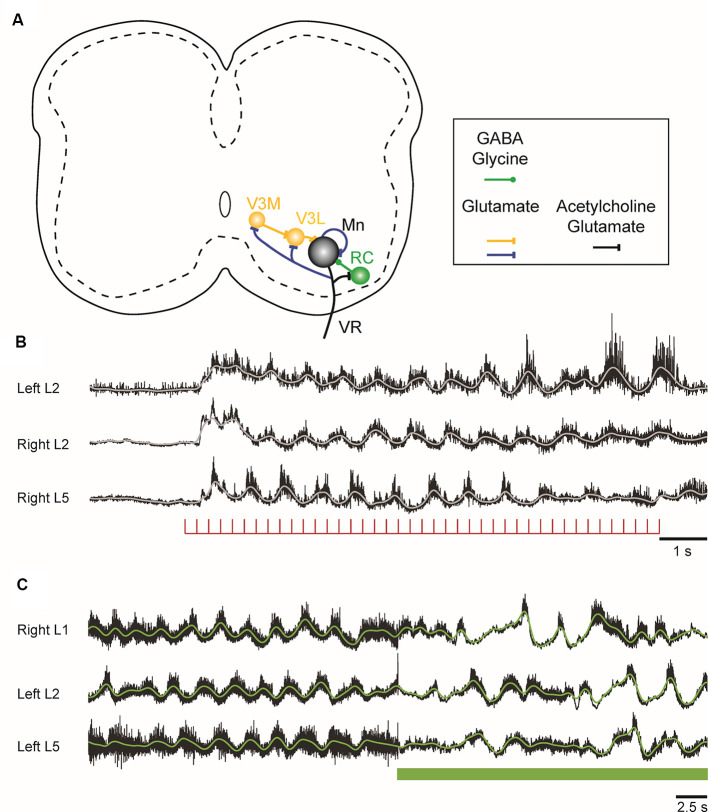
**(A)** Schematic showing the connections of motoneurons within the lumbar spinal cord of the neonatal mouse. Motoneurons project to each other, to inhibitory Renshaw cells (RCs), and to a medial (V3M) and a lateral (V3L) population of V3 interneurons. The lateral V3 population and RCs project back to motoneurons. The neurotransmitters released at the different sites are indicated in the box under the schematic. **(B)** Locomotor-like activity can be evoked in the neonatal mouse cord by a train of stimuli applied to the ventral roots. The records are the neurograms recorded from the indicated ventral roots in response to a train of stimuli applied to the right L6 ventral root. The continuous traces are the integrated records of ventral root discharge. **(C)** Optogenetic reduction of motoneuron firing slows and disrupts the locomotor-like rhythm induced by drugs. Ventral root recordings of locomotor-like activity induced by bath application of serotonin and N-methyl-D-aspartate (NMDA) on a spinal cord expressing the inhibitory opsin archaerhodopsin in cholinergic neurons. On exposure to green light (green bar), the neurons expressing the opsin are hyperpolarized, leading to a slowing and disruption of the locomotor rhythm.

Recently, new evidence has emerged, showing that motoneurons have more extensive intraspinal synaptic targets that were previously realized. In 2005, it was demonstrated that motoneurons release an excitatory amino acid—probably glutamate—in addition to acetylcholine at their central connections with RCs in the neonatal mouse spinal cord (Mentis et al., 2005; Nishimaru et al., 2005) and confirmed a few years later in both the neonate (Lamotte d’Incamps and Ascher, 2008) and the adult mouse (Lamotte d’Incamps et al., 2017). Subsequent work in neonatal and juvenile mice showed that motoneurons make powerful glutamatergic connections with each other, with the largest inputs to type F motoneurons (Figure 2A; Bhumbra and Beato, 2018). This surprising finding indicates that motoneurons release different transmitters at different axonal branches apparently contravening Dale’s principle (Dale, 1935; Eccles et al., 1954). However, it is possible that both transmitters are present at the motoneuronal terminals on motoneurons because the failure to detect cholinergic responses could be due to the absence of postsynaptic acetylcholine receptors at the synapse. In addition, motoneurons in the neonatal mouse spinal cord also project exclusively glutamatergic synapses to a class of glutamatergic, commissural spinal interneurons called V3 interneurons (Chopek et al., 2018).

In the neonatal mouse cord, stimulation of motor axons can initiate locomotor-like activity (Figure 2B; Mentis et al., 2005; Pujala et al., 2016), and optogenetic manipulations of motoneuron firing regulate the frequency of the locomotor rhythm during drug-induced locomotor-like activity (Figure 2C; Falgairolle et al., 2017). The mechanisms by which motoneurons influence the central pattern generator (CPG) are not fully understood (Falgairolle and O’Donovan, 2019a). Surprisingly, it occurs in the absence of cholinergic transmission and depends instead on glutamatergic transmission (Mentis et al., 2005; Falgairolle et al., 2017). It has been proposed that these excitatory effects of motoneuron stimulation on spinal circuitry are most simply explained by the presence of an excitatory interneuron interposed between the locomotor central pattern generator and motoneurons (Machacek and Hochman, 2006; Bonnot et al., 2009; Falgairolle et al., 2017). The glutamatergic V3 interneuronal population is clearly a candidate for such a neuron because it receives direct monosynaptic input from motoneurons. The V3 interneurons comprise a medial and a lateral group. The medial V3 neurons project to the lateral group which in turn projects back to motoneurons, thus forming a recurrent excitatory connection with motoneurons (Figure 2A; Chopek et al., 2018). The existence of this pathway probably explains earlier observations that revealed recurrent, disynaptic excitation of motoneurons in the neonatal rat (Schneider and Fyffe, 1992; Ichinose and Miyata, 1998). However, the effects of genetic silencing or elimination of V3 interneurons on the locomotor rhythm are not the same as optogenetic hyperpolarization of motoneurons. The silencing experiments show that V3 neurons are required to balance the excitatory locomotor drive to both sides of the cord (Zhang et al., 2008), whereas hyperpolarization of motoneurons slows the locomotor frequency (Falgairolle et al., 2017). Furthermore, optogenetic excitation of V3 interneurons expressing channelrhodopsin slows the locomotor-like rhythm (Danner et al., 2019), in contrast to the acceleration that occurs when motoneurons are optogenetically excited. This suggests that the V3 neurons do not mediate the excitatory effects of motoneurons on the locomotor CPG, raising the possibility that other—currently unidentified—classes of interneurons are targeted by motoneurons.

Furthermore, it is not clear if glutamate release from the VGLUT2 from motoneurons mediates the excitatory effects of motoneurons because selective elimination of VGLUT2 from cholinergic neurons—including motoneurons—has no effect on locomotor-like activity (Caldeira et al., 2017). However, because this was a chronic study, some type of compensation may have occurred to offset the absence of motoneuronal glutamatergic inputs. Alternatively, glutamate release from motoneurons may not be mediated exclusively by VGLUT2, although it is difficult to support this idea given that the glutamatergic component of the motoneuron–Renshaw synapse is abolished in the VGLUT2 knockout (Talpalar et al., 2011).

In the adult zebrafish, motoneurons have also been shown to modulate locomotion (Song et al., 2016). In this animal, motoneurons have reciprocal hybrid chemical/electrical synapses with a class of excitatory interneurons (V2a) that are believed to be important in generating the swimming rhythm (Eklöf-Ljunggren et al., 2012). Motoneuron membrane polarization can modulate transmitter release from the V2a interneurons by polarizing the V2a terminals on motoneurons. In addition, motoneuron membrane potential can directly modulate the firing of V2a interneurons and the swimming frequency. In the neonatal mouse spinal cord, this mechanism does not appear to be responsible for the effects of motoneuron activity on the rhythm because blockade of gap junctions with carbenoxolone does not attenuate the effect of motoneuron activity on the frequency of the rhythm (Falgairolle et al., 2017). Consistent with this idea, application of the α-amino-3-hydroxy-5-methyl-4-isoxazolepropionic acid (AMPA) receptor antagonist NBQX, blocked the effects of motoneuron activity on the rhythm, suggesting that motoneuronal connections to the CPG are mediated by an excitatory interneuron contacted by motoneurons (Machacek and Hochman, 2006; Bonnot et al., 2009; Falgairolle et al., 2017). Furthermore, it has also been shown that V2a interneurons do not receive synaptic or electrical inputs from motoneurons in the neonatal mouse (Bhumbra and Beato, 2018).

## Synaptic Connections of Motoneurons and Their Susceptibility to Disease

In the final section of this review, we ask if any of the input or output connections of the different motoneuron types, the extraocular motoneurons, and Onuf’s nucleus (Comley et al., 2016; Nijssen et al., 2017) are correlated with their susceptibility to disease.

Several correlations are apparent in the data of Figure 1. For example, the most resistant motoneurons are the smallest, and they receive the fewest and the least diverse synaptic inputs. Thus, within the α-motoneuron population, the number of 1a terminals progressively decreases from type FF to S and are absent on γ-motoneurons, extraocular motoneurons (Keller and Robinson, 1971), and Onuf’s nucleus (Lalancette-Hebert et al., 2016). Similarly, C-boutons are absent on extraocular motoneurons (Hellström et al., 2003; Rozani et al., 2019) and γ-motoneurons (Arvidsson et al., 1987), with a gradient of inputs from type S to type F α-motoneurons (Hellström et al., 2003). However, at least for primary afferents, their presence or absence does not seem to be associated with motoneuron cell death. For instance, although the number of VGLUT1^+^ primary afferents on motoneurons decreases before motoneuron death, their restoration does not prevent motoneuron death (Fletcher et al., 2017). In ALS, by contrast, ablation of primary afferents exerts a protective effect on α-motoneurons (Lalancette-Hebert et al., 2016). Similarly, although the least susceptible motoneurons lack C-boutons in both diseases, during the progression of ALS, C-boutons become more numerous on vulnerable α-motoneurons, and the number of cholinergic interneurons in Lamina X (presumably the source of C-boutons) increases. Although both decrease toward the end of the disease, the initial changes may reflect compensatory adaptations to maintain motoneuron excitability (Milan et al., 2015). In SMA, the number of cholinergic interneurons does not change (Powis and Gillingwater, 2016), and furthermore, when the C-boutons are restored in the ALS mouse model, they extend survival time (Lasiene et al., 2016), suggesting that the presence of C-boutons and their normal function may facilitate motoneuron survival. Collectively, these observations suggest that the distribution of primary muscle spindle afferents and C-boutons on motoneurons is probably not the factor that contributes to their vulnerability.

A more consistent association emerges when we consider the number of recurrent collaterals produced by the different motoneurons. Those with the greatest number of intraspinal collaterals (type F) are the most susceptible, and those with the fewest γ-motoneurons and extraocular motoneurons are the least (Evinger et al., 1979). About half the motoneurons in Onuf’s nucleus have no recurrent collaterals (Sasaki, 1994). This relationship may also extend to the well-known difference in the sensitivity of proximal and distal muscles to the disease process in both SMA and ALS. In the adult cat, the most distal limb muscles, including many of the foot and forepaw muscles, lack recurrent collaterals (Hörner et al., 1991; McCurdy and Hamm, 1992; Illert and Kümmel, 1999), and recurrent inhibition is much more pronounced in motoneurons innervating the muscles of the elbow than of the wrist (Hahne et al., 1988). Furthermore, motoneurons innervating the intercostal muscles have been shown to have axon collaterals and receive recurrent inhibition (Kirkwood et al., 1981; Lipski and Martin-Body, 1987), and axial motoneurons receive recurrent inhibition (Jankowska and Odutola, 1980), suggesting that they have axon collaterals projecting to RCs as do α-motoneurons. Why would the number of motoneuron collaterals be associated with disease susceptibility? Before motoneurons die, their intraspinal connections to RCs are lost (Wootz et al., 2013) and any functions associated with these connections will also be lost. For example, loss of motoneuron input to the locomotor CPG could compromise locomotion, although this could be compensated by interneurons that also influence locomotor function (Gosgnach et al., 2006; Dougherty et al., 2013; Talpalar et al., 2013; Falgairolle and O’Donovan, 2019b).

It might seem paradoxical that an absence of recurrent collaterals, and presumably Renshaw inhibition, would be associated with a protection against the disease process. Enhanced motoneuron excitability, particularly at early stages of the disease, is often proposed as one of the mechanisms contributing to pathophysiology of motoneurons (Kuo et al., 2004, 2005; Jiang et al., 2017). The Renshaw pathway exerts a powerful inhibitory effect on motoneurons (Moore et al., 2015) and would therefore be expected to temper any increases in motoneuron excitability. However, motoneurons also receive monosynaptic glutamatergic input from other motoneurons and recurrent excitation from V3 glutamatergic interneurons. If motoneuronal inputs to inhibitory RCs are lost before those to motoneurons, this would result in powerful, recurrent glutamatergic excitation of motoneurons unbalanced by recurrent inhibition particularly in the type-F population which receives the strongest excitatory input from other motoneurons (Bhumbra and Beato, 2018). This could lead to glutamate toxicity and a compensatory reduction of motoneuron excitability. Consistent with this suggestion, a reduction of motoneuron excitability in type-F motoneurons is observed to precede denervation in the SOD1-G93A and FUS-P525L mouse models of ALS (Martinez-Silva et al., 2018).

As with their central connections, type FF motoneurons have the most intramuscular synaptic connections (Burke, 1978). Because synapses are energetically demanding (Harris et al., 2012), the FF motoneurons have the highest metabolic demands (Le Masson et al., 2014) which may increase their susceptibility to the disease given that mutant SOD1 can compromise mitochondrial function (Pasinelli et al., 2004).

An alternative and complementary interpretation for the relation between the number of recurrent collaterals and disease susceptibility derives from the idea that a neuron is dependent on *all* its synaptic targets for trophic support. It is well known that during development, motoneuron survival depends on its target muscle for survival, but as the animal matures, this dependence is reduced (de la Cruz et al., 1996). What is less clear is the extent to which the functions and properties of motoneurons also depend on trophic support from their synaptic targets within the central nervous system. The recent discoveries that spinal motoneurons have novel synaptic targets within the cord mean that trophic support from these neuronal populations has necessarily been underappreciated. According to this idea, as motoneurons disconnect from their synaptic targets within the spinal cord (Wootz et al., 2013), they lose the trophic support normally provided by these targets. The motoneurons lacking recurrent collaterals would thus be resistant to this process because they presumably derive their trophic needs from other sources including the motoneurons themselves and the muscles they innervate. Consistent with this hypothesis, the disease-resistant extraocular muscles express higher levels of neurotrophins than other brainstem neurons that are sensitive to disease (Hernández et al., 2017; Silva-Hucha et al., 2017). Extraocular muscles also contain high levels of insulin-like growth factor (IGF) compared to other cranial or spinal motoneurons (Allodi et al., 2016). Remarkably, IGF-2 delivered by viruses to spinal motoneurons preserves the motoneurons and induces nerve regeneration in ALS (Allodi et al., 2016). It is not known if the different types of α-motoneuron or γ-motoneurons differ in their expression of trophic factors. γ-Motoneurons uniquely express the glial cell-derived neurotrophic factor (GDNF) receptor and require GDNF derived from the muscle spindle for their survival (Shneider et al., 2009). Unfortunately, trials of neurotrophins in humans have not been successful, but this is complicated by difficulties in delivering the molecules to neurons and because the appropriate neurotrophins may not have been discovered (for a review, see Kanning et al., 2010).

One observation that appears to contradict this hypothesis is the finding that motoneuron cell death is associated with an increase in the number of VAChT^+^ terminals on RCs in the SMNΔ7 model of SMA (Thirumalai et al., 2013). It is not known if these additional synapses originate exclusively from motoneurons. However, if they do, then this behavior differs from the loss of motoneuron terminals on RCs that precedes motoneuron cell death in the SOD1-G93A mouse model of ALS (Wootz et al., 2013). This difference in behavior may reflect the different ages at which motoneurons die in the two diseases. In the SMNΔ7 mouse model, motoneuron cell death begins in the neonatal period when motoneurons are not fully mature and may have an enhanced sprouting ability. It is possible therefore that the sprouting of motoneuron axons, which presumably occurs in the remaining motoneurons, is a characteristic of their immaturity rather than a fundamental difference between the two diseases.

## Author Contributions

MO’D and MF wrote and approved the article.

## Conflict of Interest

The authors declare that the research was conducted in the absence of any commercial or financial relationships that could be construed as a potential conflict of interest.
